# Involvement of glial P2Y_1_ receptors in cognitive deficit after focal cerebral stroke in a rodent model

**DOI:** 10.1186/1742-2094-10-95

**Published:** 2013-07-29

**Authors:** Yo Chin, Mayo Kishi, Masaki Sekino, Fukiko Nakajo, Yoshifumi Abe, Yasushi Terazono, Ohsaki Hiroyuki, Fusao Kato, Schuichi Koizumi, Christian Gachet, Tatsuhiro Hisatsune

**Affiliations:** 1 Department of Integrated Biosciences, The University of Tokyo, 5-1-5 Kashiwanoha, Kashiwa, Chiba 277-8562, Japan; 2 Department of Advanced Energy, The University of Tokyo, 5-1-5 Kashiwanoha, Kashiwa, Chiba 277-8562, Japan; 3 Bioimaging Center, Graduate School of Frontier Sciences, The University of Tokyo, 5-1-5 Kashiwanoha, Kashiwa, Chiba 277-8562, Japan; 4 Department of Mathematical Informatics, The University of Tokyo, 7-3-1 Hongo, Bunkyo-ku, Tokyo 113-8656, Japan; 5 Laboratory of Neurophysiology, Department of Neuroscience, Jikei University School of Medicine, Minato-ku, Tokyo 105-8461, Japan; 6 Department of Pharmacology, The University of Yamanashi, 1110 Shimogato, Chuo, Yamanashi 409-3898, Japan; 7 UMRS_949 INSERM, Université de Strasbourg, Etablissement Français du Alsace, Strasbourg, France

**Keywords:** Middle cerebral artery occlusion, Hippocampal neuroinflammation, Cognitive deficits, Diffusion tensor MRI, P2Y_1_ receptor

## Abstract

**Background:**

Neuroinflammation is associated with many conditions that lead to dementia, such as cerebrovascular disorders or Alzheimer’s disease. However, the specific role of neuroinflammation in the progression of cognitive deficits remains unclear. To understand the molecular mechanisms underlying these events we used a rodent model of focal cerebral stroke, which causes deficits in hippocampus-dependent cognitive function.

**Methods:**

Cerebral stroke was induced by middle cerebral artery occlusion (MCAO). Hippocampus-dependent cognitive function was evaluated by a contextual fear conditioning test. The glial neuroinflammatory responses were investigated by immunohistochemical evaluation and diffusion tensor MRI (DTI). We used knockout mice for P2Y_1_ (P2Y_1_KO), a glial ADP/ATP receptor that induces the release of proinflammatory cytokines, to examine the links among P2Y_1_-mediated signaling, the neuroinflammatory response, and cognitive function.

**Results:**

Declines in cognitive function and glial neuroinflammatory response were observed after MCAO in both rats and mice. Changes in the hippocampal tissue were detected by DTI as the mean diffusivity (MD) value, which corresponded with the cognitive decline at 4 days, 1 week, 3 weeks, and 2 months after MCAO. Interestingly, the P2Y_1_KO mice with MCAO showed a decline in sensory-motor function, but not in cognition. Furthermore, the P2Y_1_KO mice showed neither a hippocampal glial neuroinflammatory response (as assessed by immunohistochemistry) nor a change in hippocampal MD value after MCAO. In addition, wild-type mice treated with a P2Y_1_-specific antagonist immediately after reperfusion did not show cognitive decline.

**Conclusion:**

Our findings indicate that glial P2Y_1_ receptors are involved in the hippocampal inflammatory response. The findings from this study may contribute to the development of a therapeutic strategy for brain infarction, targeting the P2Y_1_ receptor.

## Introduction

Defects in hippocampal function caused by the progression of cerebrovascular disorders or Alzheimer’s disease can lead to memory loss. Identifying the molecular mechanisms underlying these cognitive deficits is a long-standing goal in neuroscience. Recent studies suggest that neuroinflammation may directly cause hippocampal network dysfunction in the aging brain [1,2]. Although neuroinflammation inducers, including beta-amyloid protein, hypoxia, and oxidative stresses, are generated in individual microenvironments [2], their effects converge in the downstream molecular and cellular cascades that transduce neuroinflammatory responses ( for example, microgliosis or astrogliosis) [3]. A strong association between such glial activation and changes in the hippocampal microenvironment, has been shown in mouse models of cerebral ischemia and Alzheimer’s disease [4,5]. The inflammatory responses generated by the activated astrocytes disturb synaptic plasticity and may cause a decline in cognitive function [6,7].

The glial neuroinflammatory response is known to involve the ADP/ATP receptor P2Y_1_, which is widely distributed throughout the central nervous system (CNS). The P2Y_1_ receptors are upregulated in glial cells in various brain regions after ischemia [8]. Thus, P2Y_1_ on astrocytes may transduce neuroinflammatory responses when activated by ATP released from damaged neurons or glial cells in various CNS disorders [9]. In addition, astrocytic P2Y_1_ activation induces the release of proinflammatory cytokines [10]. Thus, it is important to examine P2Y_1_’s role under pathological conditions. Here we used P2Y_1_-knockout (P2Y_1_KO) mice, which were recently reported to resist vascular inflammation [11,12]. We subjected the P2Y_1_KO mice to middle cerebral artery occlusion (MCAO) to assess P2Y_1_’s role in initiating the neuroinflammatory response in the affected hippocampus and the consequences of the neuroinflammation, including cognitive deficits. The rodent MCAO model causes both sensory-motor and cognitive deficits [13,14]. For example, rats subjected to MCAO perform worse than sham-operated rats in the Morris maze task [15]. Although the neural atrophy caused by MCAO is primarily observed in the striatum and cerebral cortex, previous studies have reported conflicting results regarding the occurrence of hippocampal damage following MCAO [13,15-18].

To monitor putative hippocampal microstructural changes relating to the neuroinflammation caused by MCAO, we applied a sensitive neuroimaging method, diffusion tensor MRI (DTI) [19,20]. DTI can detect microscopic structural changes that alter water diffusivity, such as the enlargement of glial cells upon their activation, in a region of neuroinflammation [21,22]. This technique previously revealed the magnitude of water diffusivity alteration in the rat hippocampus after two-vessel occlusion [23] and in the mouse hippocampus in an Alzheimer’s disease model [24,25].

In this study, we focused on the hippocampal environmental changes following the neuroinflammatory response induced by MCAO. We examined glial activation by immunohistochemistry and structural changes by DTI. The aim of this study was to discover how an alteration in the hippocampal microenvironment affects cognitive function after MCAO. We also focused on the role of the astrocytic P2Y_1_ receptor in regulating the neuroinflammatory response that affects cognitive function.

## Material and methods

### Animals

Adult male Sprague Dawley rats (280 to 300 g) and C57/BL6 mice (18 to 25 g) were from Sankyo Labo Service Corporation (Tokyo, Japan). The P2Y_1_KO (-/-) (C57/BL6 background) mice were generated previously [11]. All animals were housed in individual cages with a 12-h light/dark cycle and access to food and water *ad libitum*. Experimental procedures were carried out in accordance with animal experimentation protocols approved by the Animal Care and Use Committee of the University of Tokyo.

### Surgery

Rats and mice were anesthetized intramuscularly with 100 mg/kg ketamine hydrochloride and 25 mg/kg xylazine. Rectal temperature was monitored continuously by a thermometer and heating pads (BWT-100, Bio Research Center, Nagoya, Japan) were used to automatically maintain body temperature at 37.0 to 37.5°C.

For rat MCAO, transient focal cerebral ischemia was induced by a 90-min occlusion of the right middle cerebral artery, as described previously [26]. In brief, a 4-0 nylon monofilament (Nitcho Kogyo Co., Ltd., Tokyo, Japan) with a silicon-coated tip was introduced into the right internal carotid artery from the common carotid artery, and the filament tip was kept in place for 90 min. The filament was then withdrawn from the internal carotid artery to allow reperfusion. After good spontaneous breathing was confirmed, each animal was returned to its cage. For the sham-operated rats, the common carotid artery and the right external carotid artery were occluded.

For mouse MCAO, 8-0 nylon with an expanded (heated) tip was introduced into the right internal carotid through the external carotid stump, as described previously [27]. The infarction time was 45 min. To evaluate the infarcted area, serial coronal sections were obtained from sites +3, +2, +1, +0, -1, -2, and -3 mm from the bregma. The sections were stained with 0.5% cresyl violet and scanned on both sides using a digital camera (Camedia C-5050, Olympus Corporation, Tokyo, Japan). For the continuous administration of MRS2500 (Tocris Bioscience, Minneapolis, MN, USA) into mice, a micro-osmotic pump (model 1007D, Alzet, Palo Alto, CA, USA) filled with 100 μL MRS2500 (1 mg/mL), delivering 0.5 μL/h, was implanted intracerebroventricularly immediately after reperfusion using a Brain Infusion Kit 3 (Alzet, Palo Alto, CA, USA).

### Behavioral tests

To evaluate cognitive function, mice and rats were subjected to fear conditioning 24 h before contextual and cued tests. For conditioning, rats were placed individually in a conditioning chamber (Med Associates, Inc., St Albans, Vermont, USA) for 5 min; after 3 min they were given two tone/foot-shock pairings. A 10-s tone (80 dB, 5 KHz) preceded a 2-s foot-shock (0.75 mA) that co-terminated with the tone (60-s interstimulus interval). Mice were placed in a box for 6 minutes and given three tone/foot-shock pairings (1 mA).

For both mice and rats, the chambers were inside a sound-attenuating box equipped with a fan as white noise, to minimize the effect of outside noise. The box was cleaned with 70% isopropanol before each use. For contextual testing, freezing behavior was scored for 8 min without any stimulation. For cued testing, the animals were placed in a different context (red lighting, flat floor, and curved wall) for 5 min following the contextual test. After 3 min, a 10-s tone was delivered twice using the same protocol as in the conditioning, without the shock. The animals’ freezing behavior was scored for 3 min before the tone (pre-tone) and for 2 min after the tone was delivered (post-tone).

A 21-point behavioral scale [28] was used to evaluate the sensory-motor function of rats after MCAO. Sham and MCAO groups were prepared specifically for the daily behavioral experiments (n = 6 each). However, when mice were used for these tests, we could not evaluate their standing ability on a 45-degree slope as described by Hunter [28], because they weighed too little for the test to be executed correctly.

### Diffusion tensor imaging data acquisition

Following the behavioral tests, the rats were sacrificed at three time points: 1 week, 3 weeks, and 2 months after surgery (only at 1 week for sham-operated animals). They were perfused with phosphate-buffered saline (PBS) intracardially and decapitated. Each brain was post-fixed in 4% formaldehyde (from paraformaldehyde) in PBS (pH 7.4) for 30 min at 4°C. The fixed brain was rinsed with PBS and was then placed into the scanner within a plastic tube.

The magnetic resonance (MR) experiments on rats were performed in a 4.7 T scanner (Varian Associates, Palo Alto, CA) equipped with gradients of up to 60 mT/m. A 66-mm volume coil was used for both transmission and reception. A 2D diffusion-weighted (DW) spin-echo sequence was used with the following acquisition parameters: FOV = 30 × 30 mm^2^, image matrix = 128 × 128, slice thickness/gap = 1/0 mm, TR/TE = 3000/50 ms, NEX = 40. DW images were acquired in 12 directions with 2 b-values of 0 and 1496 s/mm^2^. The total acquisition time was 55 h.

Mice were perfused intracardially with PBS, then with 4% formaldehyde in PBS (pH 7.4), and decapitated. Each brain was post-fixed in 4% formaldehyde for at least 3 days at 4°C. The fixed brain was rinsed with PBS and immersed in Fluorinert (Sigma Aldrich, St. Louis, MO, USA) in a glass tube, because the MRI system is vertical. The mouse MR experiments were performed in a 14.1 T scanner (Bruker BioSpin, Billerica , MA, USA) equipped with gradients of up to 3000 mT/m. A 10-mm volume coil was used for both transmission and reception. A 2D diffusion-weighted (DW) spin-echo sequence was used with the following acquisition parameters: FOV = 6 × 6 mm^2^, image matrix = 128 × 128, slice thickness/gap = 0.5/0 mm, TR/TE = 2500/27 ms, NEX = 16. DW images were acquired in 12 directions with 2 b-values of 0 and 1500 s/mm^2^. The total acquisition time was 19 h.

For the DTI of both rats and mice, motion-probing gradients (MPGs) were applied as follows:

$$\begin{array}{l} g1=\frac{\sqrt{5}}{5}\left(201\right),g2=\frac{\sqrt{5}}{5}\left(012\right),g3=\frac{\sqrt{5}}{5}\left(120\right),\\ g4=\frac{\sqrt{5}}{5}\left(210\right),g5=\frac{\sqrt{5}}{5}\left(021\right),g6=\frac{\sqrt{5}}{5}\left(102\right),\\ g7=\frac{\sqrt{5}}{5}\left(20‒1\right),g8=\frac{\sqrt{5}}{5}\left(012\right),g9=\frac{\sqrt{5}}{5}\left(‒120\right),\\ g10=\frac{\sqrt{5}}{5}\left(2‒10\right),g11=\frac{\sqrt{5}}{5}\left(02‒1\right)\;\text{and}\;\\ g12=\frac{\sqrt{5}}{5}\left(‒102\right) \end{array}$$

These sample preparation and DTI data acquisition methods were based on previous research with minor modifications [19,29].

### Image and data analysis

The six independent elements of the 3 × 3 diffusion tensor were calculated from each series of DW images. The tensor was diagonalized to obtain three eigenvalues (λ_1-3_), which corresponded to the three eigenvectors (ν_1-3_). The DTI index Trace*(D)*, which is a scalar measurement of the total diffusion within a voxel, was derived from the DTI-studio software [30].

$$\text{Trace}\left(D\right)=\lambda_{1}+\lambda_{2}+\lambda_{3}$$

The mean diffusivity (MD) is a measure of the average motion of water molecules independent of directionality, which was obtained from the Trace*(D)*. For the color-coded MD maps, MATLAB (Math Works, Natick, MA, USA) was used. The MD value range was defined as 0 to 5.0 × 10^-4^ mm^2^/sec.

$$\mathrm{MD}=\frac{\lambda_{1}+\lambda_{2}+\lambda_{3}}{3}=\frac{\text{Trace}\left(D\right)}{3}$$

Regions of interest (ROIs) were semi-automatically defined in the images generated from DTI Studio, by ROI Editor [30].

To make the T-contrast map, SPM5 software (Welcome Trust Center for Neuroimaging, England, UK) running on MATLAB, was used for slice realignment and spatial normalization of the DTI data [31]. We analyzed the areas that showed a significant decrease in MD (*P* <0.001, uncorrected) at 1 week after MCAO (n = 6), compared with intact animals (n = 7).

### Immunohistochemical analysis

For fluorescence immunohistochemistry, frozen specimens were coronally sectioned with a cryostat (Microm) at a thickness of 40 μm. After being blocked in 3% donkey serum, the sections were incubated with anti-glial fibrillary acidic protein (GFAP; mouse, 1:800; Sigma Aldrich, St. Louis, MO) and anti-ionized calcium binding adaptor molecule 1 antibodies (Iba1; rabbit, 1:1000; Wako) overnight at 4°C. The secondary antibodies were Alexa 488-conjugated donkey anti-rabbit IgG (1:1,000; Molecular Probes) and Cy5-conjugated donkey anti-mouse IgG (1:200; Jackson ImmunoResearch Laboratories, Inc., West Grove, PA, USA). The stained sections were incubated with DAPI (Sigma Aldrich), and observed using a confocal laser microscope (TCS SP2; Leica Microsystems, Wetzlar, Germany). Images obtained via confocal microscopy were analyzed with ImageJ software, as reported in our previous study [32]. A custom plugin was used to automatically establish the overall fluorescence from the image (> 0.28 mm^2^) at the dentate gyrus (DG)/hilus, the CA2 and CA3 (CA2/3) regions, or the CA1 region, and to calculate the percentage of the image covered by staining.

### Statistical analysis

The data are shown as the mean ± SEM. Statistical significance was determined using Student’s *t*-test and Tukey’s multiple comparison test. *P* <0.05 indicated statistically significant differences.

## Results

### Long-lasting deficit in cognitive function after middle cerebral artery occlusion in rats

**Figure 1:**
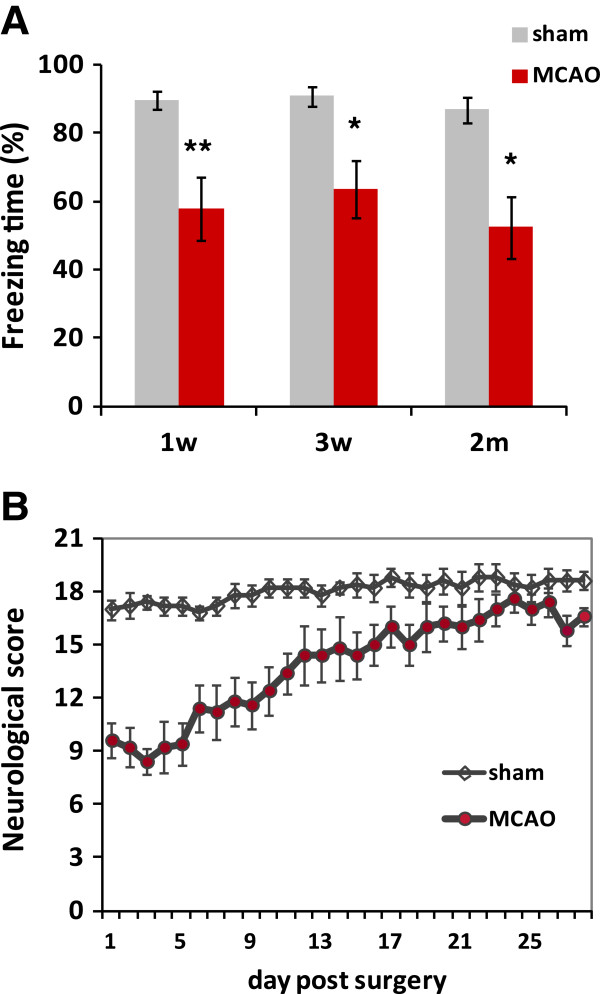
**Long-lasting cognitive decline after middle cerebral artery occlusion (MCAO) in rats. **Hippocampus-dependent cognitive function was assessed by a contextual fear conditioning test. The freezing time was measured for 8 minutes in MCAO and sham-operated rats **(A)**. Significant differences were seen at all the time points between the MCAO and sham-operated group. Daily neurological testing was conducted in a separate cohort for 4 weeks **(B)**. Most of the sensory motor function of the MCAO rats recovered within the experimental period. n = 6 in each group. Values are means ± SEM. ^*^P <0.05; ^**^P <0.01; in the *t-*test.

We tested the cognitive function of rats 1 week, 3 weeks, and 2 months after MCAO, and observed a clear decrease in the freezing time in the contextual conditioned test at all the time points tested, compared to the control rats 1 week after the sham operation (n = 6 in each group; *P* <0.05, Figure 1A). In contrast, in a cue test, we did not observe a significant difference in the freezing behavior among the groups (data not shown).

We also evaluated the time course of sensory-motor function after MCAO. A sensory-motor neurological test was conducted in a different cohort of MCAO and sham-operated rats (n = 6 in each group) every day from 1 to 28 days after the operation. As shown in Figure 1B, we detected significant recovery in the sensory-motor function of the MCAO rats, suggesting the regeneration and/or functional repair of the damaged neural networks. Taken together, these findings indicated that over the long term after MCAO, the occurrence of cognitive deficits was still a concern, despite sensory-motor recovery.

### Long-lasting hippocampal changes after middle cerebral artery occlusion in rats detected by diffusion tensor imaging

**Figure 2:**
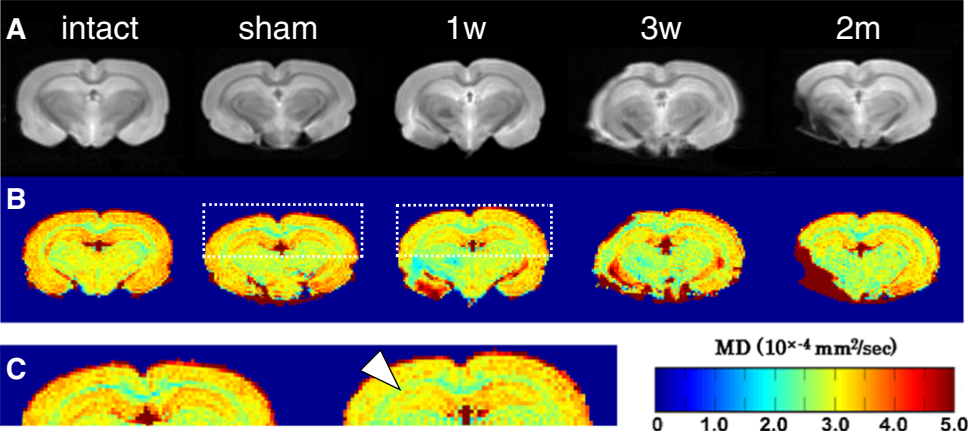
**Alteration in hippocampal mean diffusivity (MD) in middle cerebral artery occlusion (MCAO) rats visualized by MD map. **Diffusion-weighted anatomic images of the brains from the intact, sham, and 1-week, 3-weeks, and 2-months after MCAO groups **(A)**. MD color maps were created by MATLAB in the same slice for each group **(B)**. Enlarged images of the boxed hippocampal areas of the sham and 1 week after MCAO brains shown in B **(C)**. The infarcted area shows an unusually high MD value in the ipsilateral side (left) of the brain. Meanwhile, the white arrowhead indicates MD decreasing on the ipsilateral side of the hippocampus. MD values ranged from 0 to 5.0 × 10^-4 ^(mm^2^/sec), as shown in the color bar below. All slices were obtained at bregma -3.30 to -3.80 mm.

For the sensitive detection of microstructural alterations in the hippocampus of MCAO rats, we performed *ex-vivo* DTI analyses. The brain was fixed with 4% formaldehyde for 30 min at 4°C, by a slight modification of the method described by Shepherd [17]. The post-fixed brain was placed in a plastic tube without any solution and scanned in a 4.7-T animal magnetic resonance imaging (MRI) with 12-axis multi-slice DTI for 55 hours. We analyzed the change in the affected hippocampus by determining the mean diffusivity (MD) value, a type of DTI index. Examples of coronal color images of the MD values in Figure 2 show the brain from a normal non-operated rat as an intact control, the brain from a sham-operated rat 1 week after surgery as an internal control, and the brains of MCAO-treated rats 1 week, 3 weeks, and 2 months after surgery. A detailed visual inspection revealed a clear decrease in the MD value at the CA2/3 pyramidal cell layer of the ipsilateral hippocampus (indicated by an arrowhead in Figure 2C).

**Figure 3:**
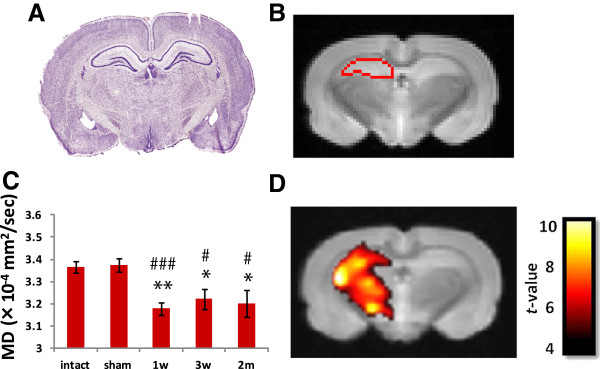
**Region of interest (ROI)-based and voxel-based morphometric (VBM) analyses of the hippocampal mean diffusivity (MD) from middle cerebral artery occlusion (MCAO) rats.** The histological images were obtained from the rat brain atlas **(A**, [33]**)**. Regions of interest (ROI) for the hippocampal area were defined semi-automatically on diffusion-weighted images by ROI Editor **(B**: bregma -3.30 to -3.80 mm**)**. The hippocampal MD of the ipsilateral side was calculated for each group **(C)**. All the post-MCAO groups showed a significant decrease. n = 6 for each group. Values are means ± SEM. *P <0.05; **P <0.01 in the *t-*test versus intact group. ^#^*P *<0.05; ^###^*P *<0.001 in the *t-*test versus sham-operated group. T-contrast map showing a significant decrease in MD value was observed in the 1 week after MCAO group compared to the intact group **(D**: uncorrected, *P *<0.001**)**. The ipsilateral hippocampus and striatum areas showed significant differences. For the groups tested at 3 weeks and 2 months after MCAO, the realignment process could not be performed by the software, because the chronic MCAO brains were too greatly altered. Color bars, *t *value (Height threshold t = 4.025).

Next, we quantitatively analyzed the change in the MD value at the affected hippocampus. For this analysis, we used DTI Studio for the MD calculation, with semi-automatic morphometric identification of the hippocampus by ROI Editor. We detected a significant reduction in the MD value of the ipsilateral hippocampus (*P* <0.05) after MCAO, compared to the intact or sham group (Figure 3C). The contralateral hippocampus did not show a significant MD decrease after MCAO (data not shown). For a more precise verification of the decreased MD value, we performed a voxel-based morphometric (VBM) analysis, as a fully automatic digital comparison of imaging data between the MCAO and sham-operated rats. The T-contrast map image revealed a significant reduction in the MD value after MCAO (Figure 3D).

### Hippocampal neuroinflammation after middle cerebral artery occlusion in rats

**Figure 4:**
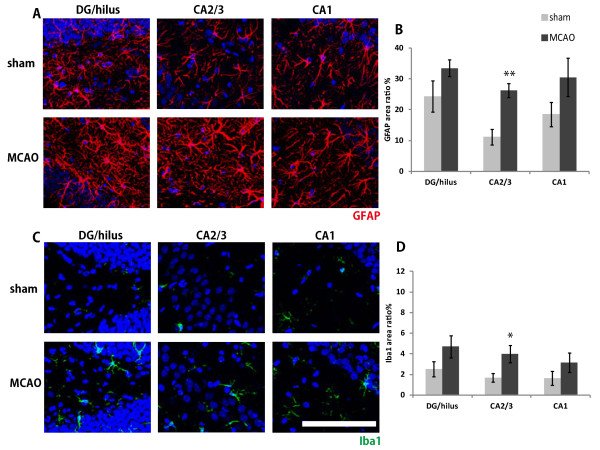
**Hippocampal neuroinflammatory gliosis after middle cerebral artery occlusion (MCAO) in rats. **Representative comparisons between 1 week post-MCAO and sham-operated rats **(A**,**C)**. Slices from each region of the hippocampus (dentate gyrus (DG)/hilus, CA2/3, and CA1) were stained with anti-GFAP and anti-Iba1 antibodies and scanned. The proportion (area ratio) of GFAP **(B) **and Iba1 **(D)** staining was calculated for each region. n = 4 in each group. Red, GFAP; Green, Iba1; Blue, DAPI. Bar represents 100 μm. Values are means ± SEM. *P <0.05; **P <0.01 in the *t-*test.

To evaluate the neuroinflammation-related cellular changes in the ipsilateral hippocampus after MCAO, we stained brain sections with anti-GFAP (a marker for activated astrocytes) and anti-Iba1 (a marker for activated microglia). As shown in Figure 4, separate subregions of the hippocampus were scanned (DG/hilus, CA2/3, and CA1). Increased activation of both astrocytes and microglia was observed in the CA2/3 region (Figure 4B, D), suggesting that neuroinflammatory responses were evoked in the hippocampus by MCAO. These alterations were also observed in the hippocampal slices 3 weeks and 2 months after MCAO (data not shown).

### Disappearance of the cognitive deficit and hippocampal neuroinflammation after middle cerebral artery occlusion in P2Y_1_KO mice

**Figure 5:**
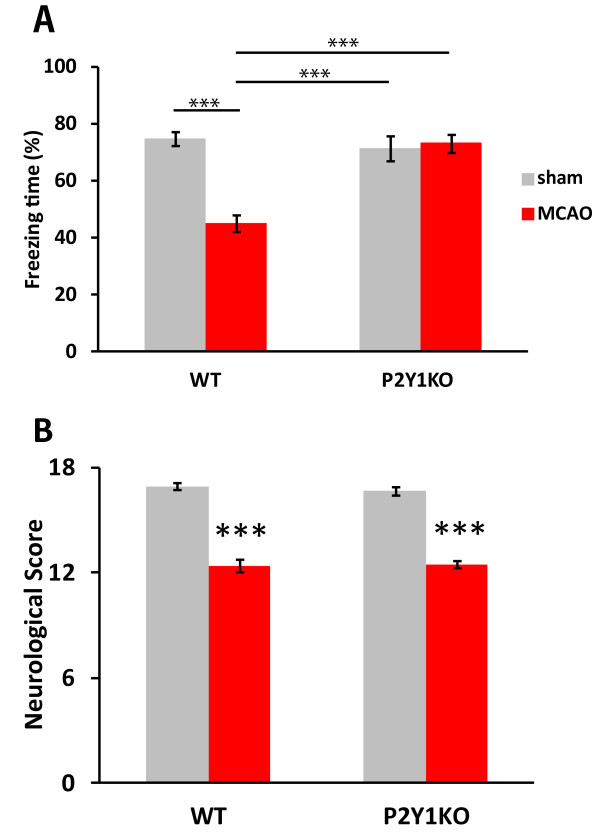
**Cognitive function was retained in P2Y**_**1**_**KO mice after middle cerebral artery occlusion (MCAO). **Contextual fear conditioning **(A)** and neurological **(B) **tests similar to the experiments with rats were performed. Both of the tests were conducted 1 week after MCAO. P2Y_1_KO mice retained their hippocampus-dependent cognitive function, while they showed sensory motor deficits similar to WT mice. WT-sham, n = 18; WT-MCAO, n = 19; P2Y_1_KO-sham, n = 14; P2Y_1_KO-MCAO, n = 14. Values are means ± SEM. **P <0.01, ***P <0.001 in Tukey’s multiple comparison test.

To examine the molecular basis of the cognitive deficits subsequent to MCAO, we used P2Y_1_KO mice and wild-type (WT) C57BL/6 mice. We prepared four groups of mice: WT-sham, WT-MCAO, P2Y_1_KO-sham, and P2Y_1_KO-MCAO. We first measured the cognitive and sensory-motor function 1 week after MCAO. In the contextual conditioning test, the WT mice showed cognitive deficits, as seen in rats. However, the P2Y_1_KO mice exhibited no decline in cognitive function after MCAO (Figure 5A). The P2Y_1_KO-sham mice exhibited cognitive function equivalent to that in WT-sham mice. The sensory-motor function was equally impaired in the WT-MCAO and P2Y_1_KO-MCAO mice (Figure 5B). The size of the infarction at the neocortex and the striatum as evaluated by Nissl staining was also equivalent between the two groups (WT: 13.6 ± 1.37%; KO: 12.1 ± 1.70% of the total brain).

**Figure 6:**
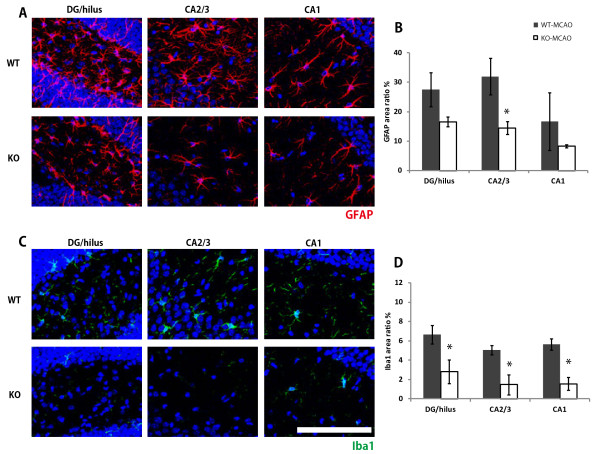
**Lack of hippocampal neuroinflammation in P2Y**_**1**_**KO mice. **Representative comparisons between WT-MCAO and P2Y_1_KO-MCAO mice 1 week after surgery **(A**,**C)**. Slices showing each region of the hippocampus (DG/hilus, CA2/3, and CA1) were stained with anti-GFAP and anti-Iba1 antibodies and scanned. The proportion (area ratio) of GFAP **(B) **and Iba1 **(D)** staining was calculated for each region. In KO mice, the GFAP expression was suppressed significantly in CA2/3, whereas Iba1 was significantly decreased in all hippocampal areas. Red, GFAP; green, Iba1; blue, DAPI. n = 4 in each group. Values are means ± SEM. *P <0.05; **P <0.01 in the *t-*test. Magnification = 40×; scale bar, 100 μm.

We stained mouse brain sections in the same manner as in the study of rats. Images from the ipsilateral hippocampus of the WT-MCAO and P2Y_1_KO-MCAO groups were evaluated (Figure 6). In the P2Y_1_KO mice, the activation of astrocytes was decreased in the CA2/3 region (Figure 6B), and significantly fewer activated microglia were seen in all the regions (Figure 6D) compared with wild-type mice, suggesting that the neuroinflammatory responses were suppressed in the hippocampus of the P2Y_1_KO mice. We also checked the background glial-activation level in the sham-operated P2Y_1_KO mice, and found no difference between the KO-sham and WT-sham groups (data not shown).

### P2Y_1_’s involvement in the cognitive decline after middle cerebral artery occlusion in mice

To examine the involvement of P2Y_1_-mediated signaling in the course of the cognitive decline induced by a focal cerebral ischemia, MCAO, we examined whether cognitive decline occurred 3 to 4 days after the operation, during a more acute phase of the ischemic injury. We chose this time point because P2Y_1_-dependent neural stem cell proliferation was not pronounced at 3 days, compared to 1 week after MCAO (data not shown); therefore, the putative P2Y_1_-dependent cognitive decline might not occur until the 1-week time point.

**Figure 7:**
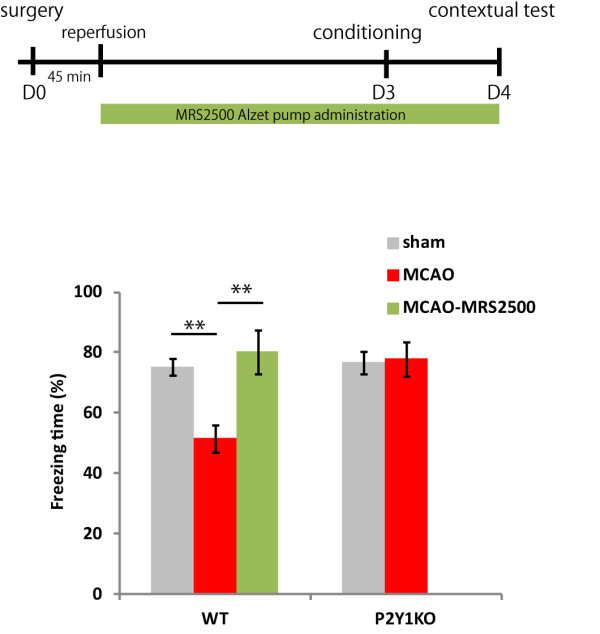
**Cognitive function in the acute phase and effect of blocking P2Y**_**1**_**. **A contextual fear conditioning test was conducted 4 days after middle cerebral artery occlusion (MCAO). The results were similar to those of the 1 week after MCAO group. The P2Y_1 _receptor antagonist MRS2500 led to remarkably maintained cognitive function. MRS2500 was administered by pump, starting immediately after the 45-min MCAO. WT-sham, n = 7; WT-MCAO, n = 10; WT-MCAO-MRS2500, n = 5; P2Y_1_KO-sham, n = 9; P2Y_1_KO-MCAO, n = 8. Values are means ± SEM. **P <0.01, in Tukey’s multiple comparison test.

For this experiment, we utilized a P2Y_1_-specific antagonist MRS2500 and prepared five groups of mice: WT-sham, WT-MCAO, WT-MCAO-MRS2500, P2Y_1_KO-sham, and P2Y_1_KO-MCAO. Contrary to our initial speculation, in the contextual conditioning tested 4 days after injury, the WT-MCAO mice showed significant cognitive deficits compared to the WT-sham mice. Interestingly, WT-MCAO mice treated with the P2Y_1_ antagonist MRS2500 by osmotic pump soon after reperfusion, did not show any cognitive decline. In addition, the P2Y_1_KO-MCAO mice exhibited cognitive function equivalent to that of the WT-sham and P2Y_1_KO-sham mice (Figure 7). Taken together, these findings suggest that P2Y_1_-mediated signaling from the time of reperfusion to 4 days after MCAO contributes to the MCAO-induced cognitive decline in mice.

### P2Y1’s involvement in the decreased hippocampal mean diffusivity value after middle cerebral artery occlusion in mice

**Figure 8:**
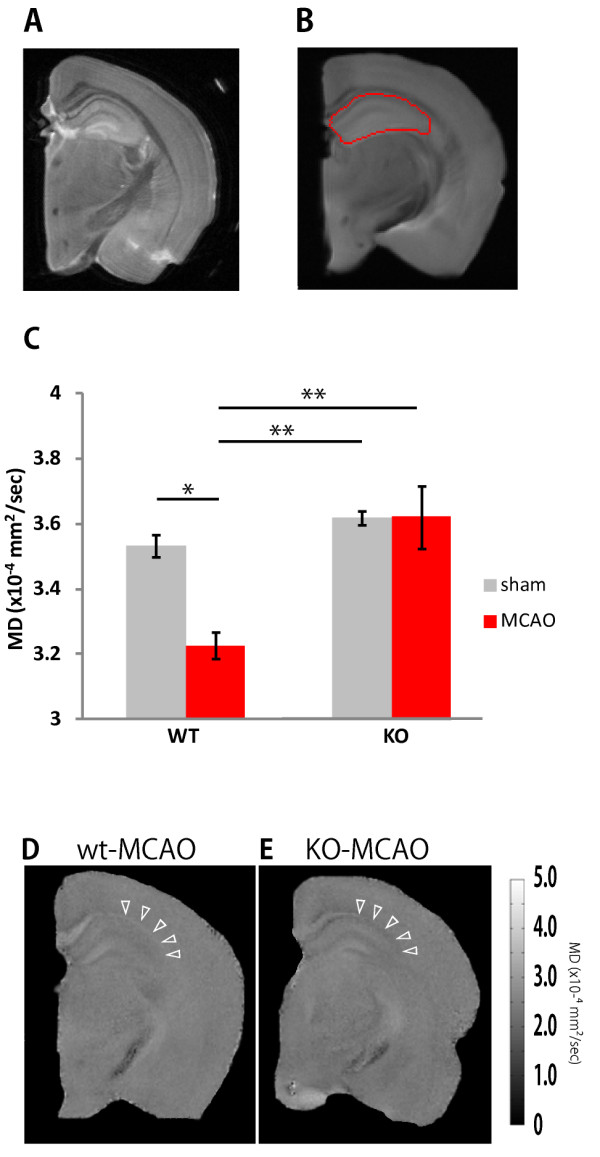
**No decrease in hippocampal mean diffusivity (MD) value after middle cerebral artery occlusion (MCAO) in P2Y**_**1**_**KO-MCAO mice. ***Ex-vivo* T2-weighted **(A) **and diffusion-weighted **(B) **images of the ipsilateral hemisphere. Regions of interest ( ROIs) for the hippocampal area were defined semi-automatically using ROI Editor **(B)**. The MD value of the ipsilateral side of the brain from four groups was calculated **(C)**. WT-sham, n = 4; WT-MCAO, n = 4; P2Y_1_KO-sham, n = 4; P2Y_1_KO-MCAO, n = 4. Values are means ± SEM. *P <0.05, **P <0.01, in Tukey’s multiple comparison test. Representative hippocampal MD maps from WT-MCAO **(D) **and P2Y_1_KO-MCAO **(E) **mice. MD maps were depicted by MATLAB. White arrowheads indicate the different MD values between the two groups at the ipsilateral side of the hippocampus. MD values ranged from 0 to 5.0 × 10^-4 ^(mm^2^/sec), shown in the bar at right. All slices were obtained at bregma -1.80 to -2.30 mm.

We next performed an *ex-vivo* DTI analysis using the brains of mice that had been used for the behavioral test shown in Figure 7. Within a day after the behavior test, the brains were fixed with 4% formaldehyde for at least 3 days at 4°C. The post-fixed brains were placed into 10-mm NMR tubes and scanned in a 14.1-T animal MRI with 12-axis multi-slice DTI for 19 hours. We analyzed the change in the affected hippocampus by determining the MD value after DTI. We detected a significant reduction in the MD value of the ipsilateral hippocampus (*P* <0.05) of the WT-MCAO mice compared to the WT-sham mice (Figure 8). In contrast, the P2Y_1_KO-MCAO brains showed a hippocampal MD value equivalent to that of the WT-sham and P2Y_1_-sham brains. A detailed visual inspection revealed a clear decrease in the MD value at the CA2/3 pyramidal cell layer of the ipsilateral hippocampus of the WT-MCAO brains, compared to that of the P2Y_1_KO-MCAO brains (indicated by arrowheads in Figure 8D, E).

## Discussion

The cognitive impairment that occurs after cerebral ischemia, especially in memory function, has recently attracted much attention [14-16,34,35]. In animal studies, MCAO disturbs cognitive function as detected by a water maze test [14,15] or a contextual conditioning test (this study). However, to our knowledge, there are few reports monitoring the chronic pathological or histological alterations in the hippocampus after MCAO. In this study, we observed impaired cognitive function, hippocampal neuroinflammation, and microstructural changes in MCAO model rats and mice. To examine the underlying mechanism affecting cognitive function, we focused on the ADP/ATP receptor P2Y_1_ expressed on activated glial cells. Our findings clearly indicated that P2Y_1_-dependent neuroinflammation contributes to the pathophysiological mechanism of the functional alteration in the hippocampus.

In this study, we successfully depicted stroke-related hippocampal microstructural alterations by an MRI method, diffusion tensor MRI (DTI). DTI has been used to detect tissue abnormalities in a variety of diseases [36-39]. *Ex-vivo* DTI has several advantages over *in-vivo* DTI, including better signal-to-noise ratio, improved spatial resolution, and fewer motion artifacts [19]. *Ex-vivo* DTI is suitable for animal studies, in which tissue can be removed for detailed evaluation, and may be superior to conventional T2-weighted imaging or immunohistochemical methods. Our results showed that MCAO caused a significant reduction in the hippocampal MD, which may indicate microstructural tissue changes owing to cell swelling or hypertrophy [36]. We assume that the stroke-related enlargement of activated astrocytes or microglial cells contributes to this putative structural change after MCAO. However, the hippocampal MD value did not recover, which would be related to the long-term deficit (2 months after MCAO) in cognitive function. In both ROI-based analyses using DTI Studio [30] and group-based analyses using SPM [31], a significant MD change between the MCAO and sham-operated animals was observed. However, we did not find a significant change in fractional anisotropy value, in contrast to a previous study [39].

It is reasonable to assume that the reduction in MD value we observed is related to the augmented neuroinflammatory response. We detected a significant elevation of the neuroinflammatory response in the CA2/3 region of the hippocampus by anti-GFAP and anti-Iba1 immunohistochemistry, and this region also showed the most significant reduction in MD value in both the MD color maps and the T-contrast map. Concurrently, we carefully examined the integrity of the CA1 pyramidal cell layer, which is the most vulnerable area in the hippocampus [40], but did not detect hippocampal neuronal death in our model (data not shown).

Our study opens the possibility that P2Y_1_-dependent inflammatory responses are associated with cognitive deficit. Recent studies have shown that ischemia increases the extracellular ATP released by damaged cells, which stimulates astrocytic P2Y_1_, resulting in high GFAP expression [10,41]. Given that GFAP upregulation occurs 12 h after reperfusion [42], we also evaluated the cognitive function at a more acute phase (3 to 4 days), and administered a P2Y_1_ antagonist, MRS2500, soon after the reperfusion. Our findings clearly showed that P2Y_1_signaling has a critical role from the time right after reperfusion through the next 4 days. During these days, it can be supposed that microglia initially reacts to ischemic damage and releases small amount of ATP which augment the P2Y1-mediated signaling on astrocytes, as reported by Pascual *et al*. [43]. Although activated astrocytes sometimes provide beneficial effects by producing cytokines that support cell regrowth [29,44], these cytokines, such as TNF-α, interleukin-6, or interleukin1-β, may also disturb synaptic plasticity, such as in LTP and cognitive function [6,7,10,45-47].

In line with this scenario, we clearly demonstrated that the blockade of P2Y_1_-mediated signaling, by either P2Y_1_KO mice or a P2Y_1_-specific antagonist, ameliorated the cognitive deficits induced by a focal cerebral stroke, MCAO. Very recently, Choo *et al*. reported that the antagonism of P2Y_1_ reduces hippocampal neuronal death and cognitive deficit after traumatic brain injury [48]. Data from our study suggest that the signaling through P2Y_1_ receptors on glial cells contributes to hippocampal neuroinflammation and cognitive deficit, and these findings may lead to new therapeutic strategies for brain infarction, targeting the P2Y_1_ receptor.

## Abbreviations

CNS: Central nervous system; DG: Dentate gyrus; DTI: Diffusion tensor imaging; DW: Diffusion-weighted; GFAP: Glial fibrillary acidic protein; Iba1: Ionized calcium binding adaptor molecule 1; MCAO: Middle cerebral artery occlusion; MD: Mean diffusivity; MPGs: Motion-probing gradients; MRI: Magnetic resonance imaging; ROI: Regions of interest; VBM: Voxel-based morphometric; WT: Wild-type.

## Competing interests

The authors declared that they have no competing interests.

## Authors’ contributions

TH, MS, and YC designed the experiments. YC, MK, FN, YA, YT, HO, FK, SK, and CG collected and analyzed data. YC and TH drafted the manuscript with the assistance of the other authors. All the authors read and approved the manuscript.
